# Identification of a panel of sensitive and specific DNA methylation markers for lung adenocarcinoma

**DOI:** 10.1186/1476-4598-6-70

**Published:** 2007-10-29

**Authors:** Jeffrey A Tsou, Janice S Galler, Kimberly D Siegmund, Peter W Laird, Sally Turla, Wendy Cozen, Jeffrey A Hagen, Michael N Koss, Ite A Laird-Offringa

**Affiliations:** 1 Norris Cancer Center and Department of Surgery and ofBiochemistry and Molecular Biology, Keck School of Medicine, University of Southern California, Los Angeles, CA 90089-9176, USA; 2 Department of Preventive Medicine, Keck School of Medicine, University of Southern California, Los Angeles, CA 90089-9176, USA; 3 Department of Surgery, Keck School of Medicine, University of Southern California, Los Angeles, CA 90089-9176, USA; 4 Department of Pathology, Keck School of Medicine, University of Southern California, Los Angeles, CA 90089-9176, USA

## Abstract

**Background:**

Lung cancer is the number one cancer killer of both men and women in the United States. Three quarters of lung cancer patients are diagnosed with regionally or distantly disseminated disease; their 5-year survival is only 15%. DNA hypermethylation at promoter CpG islands shows great promise as a cancer-specific marker that would complement visual lung cancer screening tools such as spiral CT, improving early detection. In lung cancer patients, such hypermethylation is detectable in a variety of samples ranging from tumor material to blood and sputum. To date the penetrance of DNA methylation at any single locus has been too low to provide great clinical sensitivity. We used the real-time PCR-based method MethyLight to examine DNA methylation quantitatively at twenty-eight loci in 51 primary human lung adenocarcinomas, 38 adjacent non-tumor lung samples, and 11 lung samples from non-lung cancer patients.

**Results:**

We identified thirteen loci showing significant differential DNA methylation levels between tumor and non-tumor lung; eight of these show highly significant hypermethylation in adenocarcinoma: CDH13, CDKN2A EX2, CDX2, HOXA1, OPCML, RASSF1, SFPR1, and TWIST1 (p-value << 0.0001). Using the current tissue collection and 5-fold cross validation, the four most significant loci (CDKN2A EX2, CDX2, HOXA1 and OPCML) individually distinguish lung adenocarcinoma from non-cancer lung with a sensitivity of 67–86% and specificity of 74–82%. DNA methylation of these loci did not differ significantly based on gender, race, age or tumor stage, indicating their wide applicability as potential lung adenocarcinoma markers. We applied random forests to determine a good classifier based on a subset of our loci and determined that combined use of the same four top markers allows identification of lung cancer tissue from non-lung cancer tissue with 94% sensitivity and 90% specificity.

**Conclusion:**

The identification of eight CpG island loci showing highly significant hypermethylation in lung adenocarcinoma provides strong candidates for evaluation in patient remote media such as plasma and sputum. The four most highly ranked loci, CDKN2A EX2, CDX2, HOXA1 and OPCML, which show significant DNA methylation even in stage IA tumor samples, merit further investigation as some of the most promising lung adenocarcinoma markers identified to date.

## Background

Lung cancer is expected to cause over 160,000 deaths in 2007 -killing more Americans than cancer of the prostate, breast, colon, rectum and pancreas combined [1]. Lung cancer is clinically classified into two classes: the aggressive subtype small cell lung cancer (SCLC, ~13% of cases) and non-small cell lung cancer (NSCLC, the remaining ~87%) [1]. NSCLC is histologically subdivided into four major subtypes with distinct pathological and molecular characteristics: adenocarcinoma, squamous cell lung cancer, large cell lung cancer and "other" (comprising neuroendocrine cancers, carcinoids etc.) [2]. Of these, adenocarcinoma has recently surpassed squamous cell lung cancer as the most common subtype in the United States, accounting for approximately 40% of NSCLC [3]. The incidence of lung adenocarcinoma is on the rise in many countries, in particular in women [4,5]. Adenocarcinoma is also the most common lung cancer subtype in non- and previous smokers [6].

The 5-year survival of lung cancer patients is only 15%, largely due to the fact that three quarters of lung cancer patients are diagnosed when their disease has spread regionally or distantly [7]. To make an impact on long term survival, better strategies are needed for early detection. Prior experience with chest X-ray, sputum cytology, and fiberoptic examination have failed to decrease lung cancer patient mortality, although several recent strategies show promise. Spiral computed tomography (spiral CT) is one such approach. It allows detailed imaging of the lung, and can detect very small lesions. Recent results from the Early Lung Cancer Action Project (ELCAP) indicate that this approach allows detection of early stage lung cancer [8], but in this and other studies, non-cancerous lesions far outnumber malignancies (less than 10% of lesions are cancer). In addition, it is unclear whether the early stage lung cancers identified by spiral CT represent cancers that would ultimately progress and lead to death. A recent analysis suggests spiral CT screening may not reduce lung cancer mortality [9]. Molecular analyses of plasma, sputum, and bronchial lavage fluids have also shown promise as strategies for early detection, but these methods still lack sensitivity [10]. If molecular markers with high sensitivity and specificity for cancers that will progress can be identified, such markers could be combined with spiral CT to screen high-risk individuals, allowing molecular detection and visualization of clinically relevant early lesions. This would greatly increase the chances of curative resection of lung cancer, while minimizing unnecessary and potentially life-threatening procedures in patients with benign lesions.

Of the many potential molecular markers, DNA hypermethylation – an epigenetic alteration – shows great promise. DNA hypermethylation occurs in all cancers, frequently leading to gene silencing through methylation of CpG-rich regions (CpG islands) near the transcriptional start sites of genes [11]. In lung cancer patients, such hypermethylation is quantitatively detectable in a variety of samples ranging from tumor material to blood and sputum [10]. However, to date the penetrance of DNA methylation at any single locus has not been high enough to provide great clinical sensitivity. Our focus is to increase the repertoire of sensitive DNA hypermethylation markers for lung cancer, and to compose a small panel of molecular markers that could be used to detect lung cancer with high sensitivity and specificity. Given the histopathologic, clinical and molecular differences between lung cancer subtypes, we believe that markers should be developed individually for the major histological subtypes. These markers can later be combined into a lung cancer hypermethylation panel that can be used for detection of all lung cancers.

Because of its increasing frequency and its preponderance in non- and previous smokers, we focused first on lung adenocarcinoma (AD). Here we describe our evaluation of 28 potential DNA methylation markers using primary human lung adenocarcinoma samples. To ensure that these markers detect *cancer-specific *hypermethylation changes, associated with histologically visible lung cancer (allowing surgical resection), we compared the DNA methylation profiles of the tumors with histologically normal adjacent lung tissue (AdjNTL) from lung cancer patients. We also examined non-tumor lung from non-cancer patients (NTL).

## Results

Ideal DNA hypermethylation markers for lung adenocarcinoma should show a high frequency of methylation in tumors as well as DNA methylation levels that are significantly elevated in tumor compared to non-tumor lung tissue. Environmental exposures, such as those arising from tobacco smoke, could lead to higher basal levels of methylation in non-tumor lung [12], which might affect the background signal when any resulting markers are applied to non-invasive molecular analyses of bodily fluids in the future. To ensure the identification of markers that are more highly methylated in adenocarcinoma even when compared to heavily exposed but histologically cancer-free lung, we used adjacent non-tumor lung (AdjNTL) from lung cancer cases as our cancer-free comparison. The AdjNTL sections were derived from separate, histologically verified cancer-free paraffin blocks. We also examined a number of non-tumor lung (NTL) samples from patients operated for non-cancer reasons (emphysema, lung collapse, etc.). Quantitative assessment of DNA methylation levels allows a more detailed evaluation of candidate DNA methylation markers, and of their suitability for correctly identifying a cancer vs. non-cancer sample. For this reason, we used the bisulfite conversion based real-time PCR technique, MethyLight, to measure DNA methylation in tumor and control tissues [13].

Twenty-eight loci were chosen for evaluation (Table 1). The choice of loci was based on a prescreening of 114 loci carried out on a collection of human lung cancer cell lines, including 11 adenocarcinoma cell lines (unpublished data). We also included many loci that appeared promising based on previous reports describing their DNA methylation in lung cancer or other cancers, so that all markers of interest could be compared on one set of tissues using a single technique and platform. Among others, the 28 loci included CpG islands in the promoters of tumor suppressor genes and genes with important roles in cell cycle regulation, DNA repair, and apoptosis (Table 1).

**Table 1:** Gene name and function of the 28 loci studied

**HUGO acronym**^a^	**Gene Name**^b^	**Function**^c^
APC	adenomatosis polyposis coli	Tumor suppressor.
ATM	ataxia telangiectasia mutated	Tumor suppressor. DNA damage and cell cycle control.
CDH1	cadherin 1, type 1, E-cadherin (epithelial)	Involved in cell-cell adhesions, mobility and proliferation.
CDH13	cadherin 13, H-cadherin (heart)	Cell-cell adhesions.
CDKN2A EX2	cyclin-dependent kinase inhibitor 2A (melanoma, p16, inhibits CDK4)	Tumor suppressor. Cell cycle control. Involved in proliferation and apoptosis.
CDKN2B	cyclin-dependent kinase inhibitor 2B (p15, inhibits CDK4)	Cell cycle control.
CDX2	caudal type homeobox transcription factor 2	Transciptional regulation. Involved in differentiation.
CHFR	checkpoint with forkhead and ring finger domains	Cell cycle control. Involved in signaling.
CYP1B1	cytochrome P450, family 1, subfamily B, polypeptide 1	Electron transport pathway. Involved in development.
ESR1	estrogen receptor 1	Nuclear hormone receptor. Involved in the regulation of gene expression and affect proliferation and differentiation.
HMGA1	high mobility group AT-hook 1	Involved in the transcription regulation.
HOXA1	homeobox A1	Transcription factor. Involved in development.
LZTS1	leucine zipper, putative tumor suppressor 1	Involved in the regulation of cell growth. Cell cycle control and proliferation. May act as tumor suppressor.
MGMT	O-6-methylguanine-DNA methyltransferase	DNA repair.
MT1A, MT2A	metallothionein 1A, 2A	Bind heavy metals.
OPCML^d^	opioid binding protein/cell adhesion molecule-like	Involved in cell contact
PGR	progesterone receptor	Involved in the regulation of gene expression and cellular proliferation and differentiation.
PTEN	phosphatase and tensin homolog	Tumor suppressor. Involved in cell cycle progression and cell survival. Involved in cell migration and cell spreading.
RASSF1	Ras association (RalGDS/AF-6) domain family 1	Potential tumor suppressor. Invovled in apoptosis, proliferation, cell cycle progression.
SFRP1, SFRP4, SFRP5	secreted frizzled-related protein 1, 4, 5	Role in regulating cell growth and differentiation and proliferation. Involved in development.
SLC6A20	solute carrier family 6 (proline IMINO transporter), member 20	Sodium- and chloride-dependent transporter.
SOCS4	suppressor of cytokine signaling 4	Involved in signal transduction.
SYK	spleen tyrosine kinase	Involved in B cell response.
TWIST1	twist homolog 1 (acrocephalosyndactyly 3; Saethre-Chotzen syndrome) (Drosophila)	Transcription factor. Involved in differentiation.
VHL	von Hippel-Lindau tumor suppressor	Involved in transcriptional repression.

^a^Human Genome Organization nomenclature

^b^Approved gene name from Human Genome Organization website

^c^Gene function from GeneCards website

^d^MethyLight amplicon also targets the HNT CpG island

**Figure 1:**
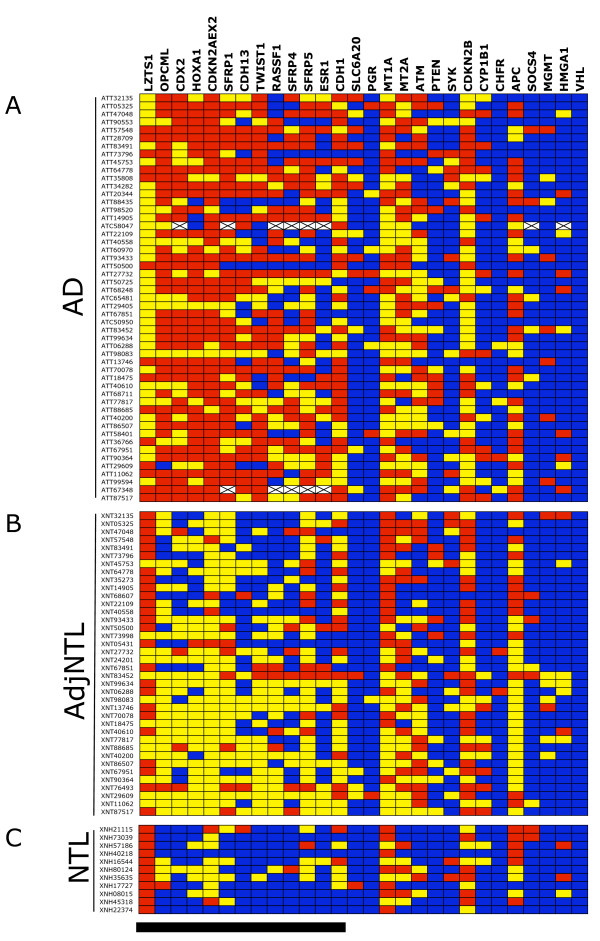
Graphic representation of PMR values obtained for 28 loci in AD (A), AdjNTL (B) and NTL (C). Samples are indicated at the left, loci at thetop. PMR values have been categorized as colored boxes denoting no detectable DNA methylation (blue), DNA methylation below the median of all positive samples of each locus (yellow), and DNA methylation equal to or above the median (red). The black bar at bottom indicates loci showing statistically significant differences in DNA methylation levels between tumor and non-tumor lung.

The results of the DNA methylation analyses for the 28 loci in 51 AD, 38 AdjNTL and 11 NTL samples are shown in Fig. 1. DNA methylation, expressed as the percentage methylated reference (PMR [14]) is visualized by color coding. Comparison of AD in Fig. 1 panel A with AdjNTL in panel B shows that a number of loci are more heavily methylated in AD. The effect appears to be even more pronounced when AD is compared to NTL from non-cancer patients. Although the exposure history of these NTL samples is unknown, their generally lower DNA methylation levels emphasize that these samples may not be the best controls when searching for loci that show cancer-specific hypermethylation. Interestingly, for one locus, LZTS1 (leftmost locus in Fig. 1), the DNA methylation pattern appeared to be reversed; NTL showed the highest level of DNA methylation, while AD samples were least methylated.

**Figure 2:**
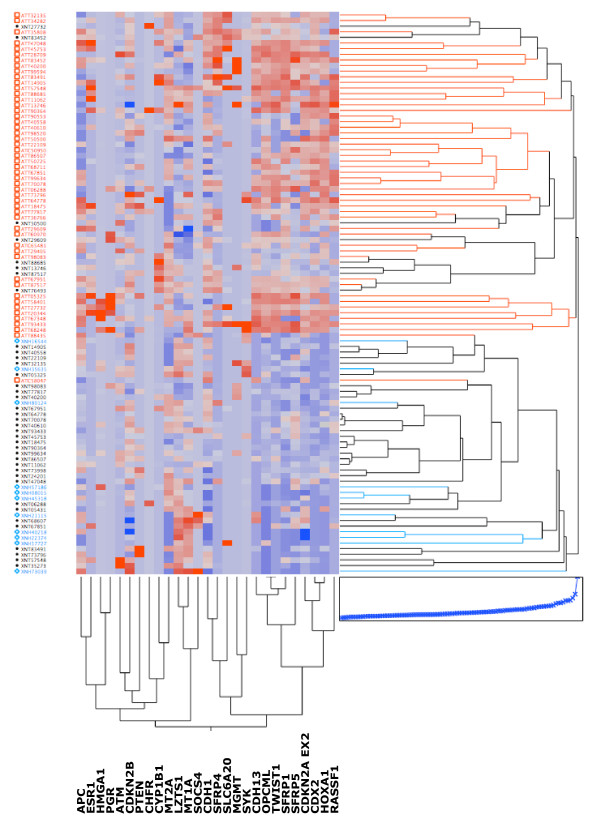
Two-dimensional hierarchical clustering of samples and loci based on DNA methylation data. In the center, DNA methylation levels are indicated by a color gradient, with the highest DNA methylation levels for each locus indicated in red and the lowest in deep blue. The Ward hierarchical clustering method was used to categorize between cancer and non-tumor samples. Sample IDs are indicated on the left, with AD samples in red, AdjNTL samples in black, and NTL samples in blue. The relationship of samples is indicated at right in the same color schematic as the labels. At bottom, the relationship of the loci is indicated. Note that all eight of the most significant loci cluster at bottom right.

We applied two-dimensional hierarchical clustering to examine the relationship between the loci and the tumor and non-tumor lung samples (Fig 2; VHL was omitted because it showed no DNA methylation in any samples). All but one of the tumor samples clustered together in a major branch of the dendrogram, while the majority of non-tumor lung samples grouped in a separate cluster. Nine loci, CDH13, SFRP1, OPCML, TWIST1, SFRP5, CDKN2A EX2, CDX2, HOXA1 and RASSF1, clustered together (bottom right), showing heavier DNA methylation in the tumor samples.

We next analyzed the statistical significance of the differences in DNA methylation levels for individual markers and different combinations of tissue samples (Table 2): AD vs. all NTL samples, AD vs. AdjNTL, and AD vs. *paired *AdjNTL (32 of the 38 AdjNTL samples were derived from the AD patients in Fig. 1A). The paired AdjNTL form an exquisite control for the cancer-specific nature of the observed DNA methylation changes, as each of these samples conforms to its tumor sample in patient age, environmental exposure, and genetic background. To avoid assigning statistical significance to spurious associations, we incorporated a multiple comparisons threshold for those loci that at time of analysis lacked any prior data suggesting they might be hypermethylated in lung adenocarcinoma (Table 2, before-last column, [15] see Materials and Methods for details). Thirteen of the analyzed loci showed statistically significant differences in DNA methylation when AD samples were compared to all NTL samples: OPCML, CDX2, HOXA1, CDKN2A EX2, SFRP1, CDH13, TWIST1, LZTS1, RASSF1, SFRP4 and 5, ESR1, and CDH1. All of these except CDH1 remained significant when AD samples were compared to AdjNTL, while all except LZTS1 remained significant in the comparison of AD to paired AdjNTL. Because DNA methylation of LZTS1 is reduced in tumors it is not a candidate for a positive lung adenocarcinoma marker and it was not studied further at this time. APC methylation was found to be statistically significantly different only when paired tumor and non-tumor lung samples were compared. This suggests that basal DNA methylation is high but variable at this locus; elevated DNA methylation in tumors is likely masked by interpatient variability and only becomes visible when samples from the same patient are compared. Indeed, Waki and coworkers have observed frequent DNA methylation of APC in non-cancer lung and other organs [16].

**Table 2:** Frequency and median PMR values of AD, Adj NTL and NTL tissues for 28 loci

**HUGO**^a^	**Frequency**^b^			**Median**^f^			**p-value**^g^				
	**AD**^c ^ **n = 51**	**Adj NTL**^d ^ **n = 38**	**NTL**^e ^ **n = 11**	**AD** ** n = 51**	**Adj NTL** **n = 38**	**NTL** **n = 11**	**AD vs All NTL**	**AD vs AdjNTL**	**AD vs matched NTL**	**BH-MC Threshold**^h^	**Importance Measure**^i^
			
OPCML	98	79	36	107.15	8.93	10.02	**9E-15**	**8E-13**	**2E-10**	0.0025	7.38
CDX2	100^j^	66	9	43.97	3.40	0.12	**4E-13**	**2E-10**	**8E-10**	0.0050	3.49
HOXA1	94	71	36	160.00	4.90	0.12	**2E-12**	**2E-10**	**2E-10**	N/A	8.52
CDKN2A EX2	100	100	82	191.07	46.17	27.39	**6E-12**	**4E-10**	**1E-10**	N/A	5.80
SFRP1	94^k^	87	36	132.93	10.01	4.78	**1E-10**	**2E-08**	**1E-09**	0.0075	2.62
CDH13	78	45	0	39.65	4.95	78.05	**4E-08**	**9E-07**	**2E-08**	N/A	2.14
TWIST1	82	66	9	392.16	6.87	8.03	**1E-07**	**8E-06**	**1E-07**	0.0100	2.77
LZTS1	100	100	100	107.75	170.95	210.95	**5E-06**	**0.0003**	0.0262	0.0125	1.52
RASSF1	69^k^	58	9	92.53	0.86	5.45	**6E-05**	**0.0010**	**1E-07**	N/A	1.83
SFRP4	67^k^	42	9	3.25	1.03	8.71	**0.0005**	**0.0052**	**0.0086**	0.0150	0.39
SFRP5	90^k^	92	45	14.38	5.78	4.59	**0.0005**	**0.0095**	**0.0010**	0.0175	0.72
ESR1	49^k^	32	9	5.72	1.29	0.63	**0.0049**	**0.0283**	**0.0007**	N/A	0.33
CDH1	94	89	55	17.62	12.74	10.22	**0.0092**	0.0686	**0.0151**	N/A	0.51
SLC6A20	25	11	9	7.81	0.38	154.45	0.0426	0.0569	0.0244	0.0200	0.10
PGR	14	5	0	59.92	6.95	N/A	0.0850	0.1753	0.4375	0.0225	0.08
MT1A	100	100	100	104.45	108.58	112.44	0.2276	0.4283	0.7422	0.0250	0.84
MT2A	88	76	55	12.31	12.22	15.48	0.2650	0.4568	0.4622	0.0275	0.37
ATM	75	68	27	0.16	0.19	0.03	0.2954	0.9498	0.5131	0.0300	0.07
PTEN	29	26	0	1.21	0.85	N/A	0.3319	0.7976	0.8077	0.0325	0.07
SYK	29	24	18	0.42	0.15	3.34	0.4314	0.5002	0.8904	0.0350	0.07
CDKN2B	98	97	91	8.29	10.05	8.48	0.4400	0.2422	0.5335	0.0375	0.44
CYP1B1	29	26	18	1.80	1.26	0.13	0.4481	0.6565	0.1454	0.0400	0.11
CHFR	8	5	0	0.49	1.65	N/A	0.4560	0.6677	1.0000	0.0425	0.04
APC	80	97	82	15.10	4.60	10.81	0.5999	0.5923	**0.0394**	N/A	1.89
SOCS4	12^j^	13	18	0.25	1.70	85.45	0.6040	0.7637	0.3125	0.0450	0.13
MGMT	16	18	0	224.53	9.22	N/A	0.7141	0.8988	0.2031	N/A	0.07
HMGA1	20^j^	18	27	0.19	0.02	0.12	0.9642	0.7645	0.6221	0.0475	0.00001
VHL	0	0	0	N/A	N/A	N/A	1.0000	1.0000	1.0000	0.0500	0

^a ^HUGO, Human Genome Organization nomenclature sorted by AD vs All NTL p-value with the most significant at the top.

^b^Percentage of samples with positive methylation value.

^c^AD, Adenocarcinoma

^d^Adj NTL, Adjacent non-tumor lung from adenocarcinoma patients

^e^NTL, Non-tumor lung from non-cancer patients

^f^Median percent methylated reference calculated from positive methylation values

^g^Statistically significant numbers are highlighted in bold; AD vs. all NTL and AD vs. AdjNTL: Wilcoxon rank sum test; AD vs. matched NTL: Wilcoxon signed rank test

^h^BH-MC threshold, Benjamini- Hochberg multiple comparison threshold p-value

^i^Importance Measure based on random forest analysis

^j^n = 50

^k^n = 49

**Figure 3:**
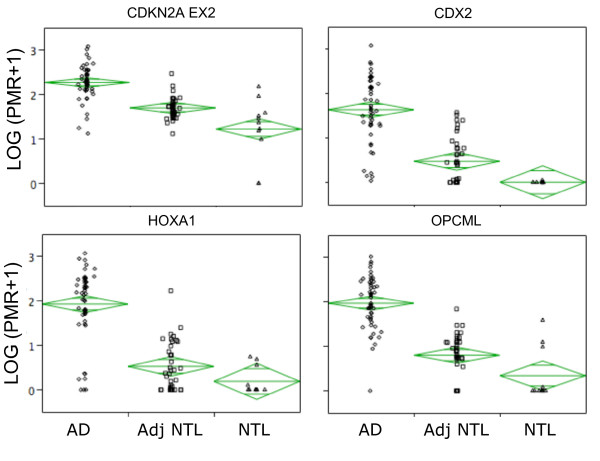
The distribution of PMR values by group. Log-transformed PMR values for AD, AdjNTL and NTL are shown. The mean is shown by the wide horizontal line, and the top and bottom of the diamond indicate a 95% normal confidence interval for the sample mean.

**Figure 4:**
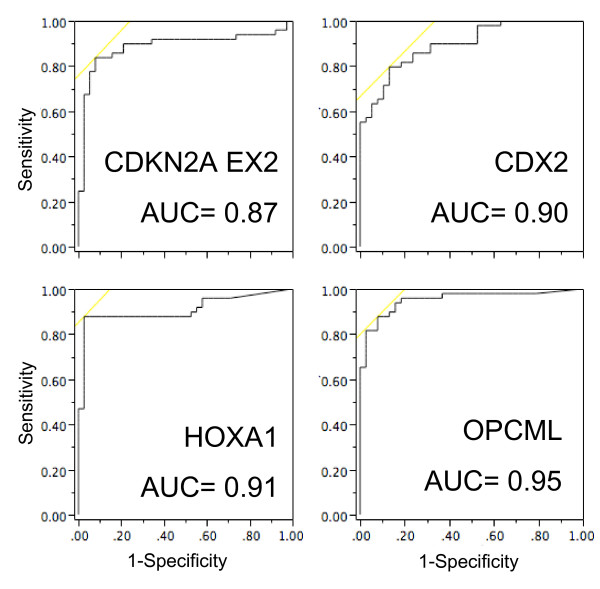
Receiver operating characteristic curves for the four top markers. All AD and AdjNTL lung samples for which there was complete DNA methylation data were used for the analysis.

Of the thirteen significant loci, OPCML, CDX2, HOXA1, CDKN2A EX2, SFRP1, CDH13, TWIST1 and RASSF1 show considerable promise as cancer-specific DNA methylation markers, exhibiting highly significant hypermethylation in tumors compared to paired non-tumor tissues (p ≤ 1 × 10^-7^, Table 2). All eight of these loci grouped together in the hierarchical clustering (Fig. 2). The ability of the top four candidates, CDKN2A EX2, CDX2, HOXA1 and OPCML (all p < 1 × 10^-9^), to individually identify lung cancer samples was next evaluated. Fig. 3 shows the distribution of PMR values in the examined sample collection. Note that for all four markers, the mean value in non-tumor lung from *non-cancer *patients is lower than that of adjacent non-tumor lung from *lung cancer *patients. This emphasizes the importance of using histologically normal tissue adjacent to lung cancer for comparison; such tissue may show higher basal DNA methylation levels while appearing histologically normal, and should be used for comparison with lung cancer tissue to ensure identification of *cancer-specific *markers. While all four markers show increased DNA methylation in adenocarcinoma compared to adjacent non-tumor tissue, the spread of DNA methylation levels differs, which would affect their sensitivity and specificity in future detection strategies. The marker potential of quantitative markers is frequently presented in the form of a receiver operating characteristic (ROC) curve, in which sensitivity vs. 1-specificity at all possible cut-off values is plotted. While these DNA methylation markers are ultimately intended for the non-invasive analysis of patient bodily fluids, a preliminary indication of their potential to sensitively and specifically detect cancer could be obtained by plotting ROC curves using the PMR values from the tumor vs. adjacent non-tumor samples. Fig. 4 shows that the area under the curve (AUC, and indicator of marker performance that would be 1 for a marker showing 100% specificity and sensitivty) is 0.87–0.95 for the four top loci.

Despite the promising AUC values, the sensitivity and specificity of these top four markers, used *individually *and determined using the current sample collection in a five-fold cross-validation, was limited: 67–86% and 74–82% respectively. This supports the notion that DNA hypermethylation markers are best used in the form of a panel. Because of the costs associated with quantitative molecular analyses, it would be important to limit the number of markers included in the panel. To determine which combinations of markers would be most effective to correctly identify tumor vs. non-tumor samples, we fit a random forest classifier to the data set, using 87 samples and 28 variables (2 AD samples with missing PMR data were omitted, resulting in 49 AD vs. 38 AdjNTL). Using bootstrap samples of the data, we grew a forest of 30,000 trees. Splits were determined using a random sample of five variables and trees were grown until there was only one observation in each leaf. Utilizing all 28 loci, we estimated a sensitivity of 92% and a specificity of 95%. Using the Gini index from the random forest classifier (last column, Table 2) to measure locus importance, we restricted our analysis to the most highly ranked variables. Reducing the locus number to 13 did not affect sensitivity and specificity, and limiting our markers to the top-ranked four (HOXA1, OPCML, CDKN2AEX2 and CDX2, which were also the most significant based on our statistical analysis) resulted in a sensitivity of 94% and a specificity of 90%. Thus, these four markers appear to be highly promising DNA hypermethylation markers for development into non-invasive molecular markers of lung adenocarcinoma, through examination of DNA shed into bodily fluids such as sputum, bronchioalveolar lavage, or blood.

For candidate hypermethylation markers of lung adenocarcinoma, two important questions arise. First, are these markers hypermethylated in cancer samples irrespective of the subject's age, gender and racial/ethnic background? And secondly, are these markers hypermethylated even in the earliest stages of lung adenocarcinoma? While the population analyzed in the current study is small, we reasoned that an indication of the potential of our top four markers to broadly identify lung adenocarcinoma might be obtained. To address the first question, we assessed correlations to age and determined whether each of the four markers showed statistically significant hypermethylation in tumor vs. adjacent normal tissues in men, women, and all four racial/ethnic groups. We found no correlation of methylation of CDKN2A EX2, CDX2, HOXA1 and OPCML with the age. In addition, all four markers remained significantly hypermethylated in tumor vs. AdjNTL when subjects were stratified by gender or by ethnic group (p < 0.05) (Table 3). The only exception was CDKN2A EX2 methylation in Asian subjects (p = 0.11), which may be related to the small sample size but will need to be further explored.

**Table 3:** Performance of top four markers in samples based on gender, race/ethnicity and stage

	Median PMR, Tumor Tumor	Median PMR, AdjNTL	p-value^a^
**GENDER**			
**Male**	**n = 28**	**n = 19**	
CDKN2A EX2	199.44	51.88	5.0E-06
CDX2	28.40	4.78	2.0E-05
HOXA1	139.51	5.06	4.6E-05
OPCML	76.62	11.27	1.6E-06
**Female**	**n = 14**	**n = 10**	
CDKN2A EX2	189.16	38.64	1.3E-04
CDX2	114.71	3.15	4.0E-06
HOXA1	188.13	1.34	3.1E-06
OPCML	154.32	5.36	3.1E-06
**RACE**			
**White Hispanic**	**n = 14**	**n = 10**	
CDKN2A EX2	165.47	37.52	6.0E-04
CDX2	39.49	1.98	2.0E-04
HOXA1	52.98	1.75	7.7E-03
OPCML	142.56	8.17	6.0E-04
**White Non-Hispanic**	**n = 14**	**n = 10**	
CDKN2A EX2	314.35	38.00	5.0E-04
CDX2	110.73	4.78	0.001
HOXA1	231.66	4.90	4.6E-05
OPCML	179.79	6.54	4.7E-05
**Black**	**n = 11**	**n = 7**	
CDKN2A EX2	194.91	51.88	0.015
CDX2	129.96	9.11	0.024
HOXA1	128.52	6.16	0.011
OPCML	91.15	14.29	0.005
**Asian**	**n = 6**	**n = 4**	
CDKN2A EX2	145.56	39.81	*0.11*
CDX2	24.28	1.78	0.023
HOXA1	117.49	0.99	0.014
OPCML	88.81	4.67	0.014
**STAGE**^b^			
**Stage IA**	**n = 12**	**n = 12**	
CDKN2A EX2	182.57	47.28	4.9E-04
CDX2	99.71	3.15	4.9E-04
HOXA1	143.06	3.25	0.001
OPCML	189.43	7.65	4.9E-04
**Stage IB**	n = 6	n = 6	
CDKN2A EX2	190.67	61.00	0.031
CDX2	20.62	13.36	*0.31*
HOXA1	78.98	2.15	*0.063*
OPCML	76.62	15.16	*0.063*
**Stage IIA/IIB/IIIA**^c^	n = 10	n = 10	
CDKN2A EX2	208.05	39.81	0.01
CDX2	83.22	1.78	0.002
HOXA1	180.18	6.16	0.002
OPCML	178.54	8.25	0.002

^a^p-value calculated by Mann-Whitney for gender and race and Wilcoxon signed rank test for stage, in which paired adjacent samples were used. Italics: p > 0.05; ^b ^Paired adjacent samples; c Only one paired sample was available for stages IIA and IIIA and none for IIIB, hence IIA/IIB/IIIA were pooled.

To address the second question, we determined whether each of the four markers was significantly hypermethylated in early and later stage tumors, using paired samples (Table 3). We examined stages IA and IB individually, but grouped stages IIA, IIB, and IIIA (only one paired sample was available for IIA and IIIA, and none for stage IIIB). Importantly, all four markers were significantly hypermethylated in stage IA cancers. Only CDKN2A EX2 hypermethylation was significant in stage IB tumors, but this could be due to the small number of paired samples (n = 6). All markers were also significantly hypermethylated in later stage lung adenocarcinoma (Stages IIA-IIIA). These analyses indicate that the top four markers show high potential for identification of lung adenocarcinoma, even in its earliest stages, an important characteristic if these markers are to be used for early detection.

To determine whether any of the four top markers might have prognostic implications, we determined whether there was any relationship between their DNA methylation level and survival. We found no significant association between DNA methylation and survival for the four loci, or any of the other 24 loci studied (data not shown).

## Discussion

Based on the results of our analyses, four loci that are very strong candidates for a DNA methylation panel aimed at early lung adenocarcinoma detection have been identified: CDKN2A EX2, CDX2, HOXA1 and OPCML. CDNK2A, also referred to as *p16*^*INK4a*^, encodes an important cell cycle regulator that is frequently inactivated in cancer. CDKN2A is one of the first tumor suppressor genes found to be methylated in a variety of cancers, including lung cancer [17]. It is one of the most widely studied hypermethylated loci, and methylation of its promoter CpG island appears to be a very early event in the development of non-small cell lung cancer (recently reviewed in [10]). In fact, methylation of the CDKN2A promoter CpG island has been observed in the sputum of subjects at risk for lung cancer 3 years prior to diagnosis [18] and in the sputum of asymptomatic heavy smokers [19]. A recent analysis of prospectively collected sputum showed CDKN2A methylation in 39% of cases and 25% of controls; methylation of this gene was associated with an elevated risk of lung cancer [20]. It is thought that DNA methylation observed in the sputum is indicative of field cancerization of the airways and not necessarily a symptom of a present cancer [20]. Our goal was to identify cancer-specific markers, not risk markers. We had evaluated DNA methylation of the CDKN2A promoter CpG island as a cancer indicator, but found substantial DNA methylation in AdjNTL, and no significant difference between AdjNTL and cancer (data not shown). Based on the cancer-specific hypermethylation of the CDKN2A exon 2 CpG island observed in colorectal and bladder cancers [21,22], we tested this downstream island instead. We established that its level of DNA methylation is a strong indicator of lung adenocarcinoma. While substantial methylation at the exon 2 CpG island is detected in histologically normal AdjNTL, by comparison, DNA methylation in adenocarcinoma is highly significantly elevated (p ≤ 1 × 10E-10). The detection of CDKN2A methylation in a high fraction of lung cancer patient plasma samples bodes well for its application to non-invasive detection [23]. Two groups reported an association of DNA methylation of CDKN2A with poor survival in adenocarcinoma/NSCLC patients [24,25], while Divine et al (2005), like us, reported no such association [12]. The differences between the obtained results might be due to the examination of a different CpG island or a different population.

DNA methylation of HOX genes, encoding homeobox transcription factors involved in embryogenesis and differentiation, had recently been observed in lung adenocarcinoma and squamous cell lung cancer. In an analysis of eight adenocarcinomas and matching adjacent lung, substantial DNA methylation of the HOXA and D clusters was observed [26]. Five cancer samples showed DNA methylation of HOXA1, while only one AdjNTL sample was methylated at this locus. In a different study, analysis of a stage I adenocarcinoma and squamous cell lung carcinoma showed DNA methylation of the HOX clusters, and examination of the HOXA and D clusters in more detail in squamous cell cancers and control tissue indicated a DNA methylation frequency of 45–80% for HOXA7-9, but methylation of HOXA1 was limited [27]. Neither of these studies examined a large number of adenocarcinomas, nor were quantitative techniques used. Here we demonstrate that HOXA1 is a very promising DNA methylation marker for lung adenocarcinoma. We have also observed DNA methylation of additional HOX genes (unpublished studies), but HOXA1 appears to be particularly informative.

OPCML, encoding an opioid-binding cell adhesion molecule, has been shown to be frequently methylated in ovarian cancer [28]. Given that opioids have demonstrated growth inhibitory and pro-apoptotic effects in lung cancer cells [29-31], it is perhaps not surprising that the OPCML promoter CpG island might be a target for DNA methylation in lung cancer. Very recently, high throughput DNA methylation profiling of 11 lung adenocarcinomas and control lung identified a number of CpG dinucleotides methylated in the cancer samples [32]. One probe identified DNA methylation in the area covered by the OPCML probe used here. Although the OPCML locus was not studied in detail in the Bibikova study, the observed methylation supports the idea that OPCML is a strong candidate marker in lung adenocarcinoma.

CDX2, another homeobox transcription factor, had been described to be methylated in squamous esophageal cancer [33] and colorectal carcinoma [34], but to our knowledge, its DNA methylation in lung cancer has never been examined. We find it to be methylated in 100% of lung adenocarcinomas, showing a 10-fold higher median methylation than AdjNTL tissue (Table 2).

Because our primary goal is marker development, here we focused only on whether loci showed consistent hypermethylation. Whether or not this hypermethylation results in gene inactivation is not relevant for the use of these loci as DNA methylation markers, and was not determined. However, the biological consequences of the observed hypermethylation would also be worth investigating. While each of the four top-ranked loci is of interest as a DNA methylation marker, it is as a *panel *that they promise to be most powerful. To our knowledge, we are the first to examine CDKN2A EX2, CDX2, HOXA1 and OPCML in combination. The fact that this marker set allows identification of cancer specimens in the current tissue collection with a substantially higher sensitivity and specificity than any previously identified single markers underlines the importance of developing suitable marker panels.

## Conclusion

From a starting panel of 28 DNA methylation loci, we have identified 13 that show statistically significant methylation differences between lung adenocarcinoma and non-cancer lung tissue. Of these, 8 show highly significant differences. The four most significant markers also ranked as the top four to be used in a marker panel, as determined by a random forest approach. Thus, we suggest that CDKN2A EX2, CDX2, HOXA1 and OPCML are the top candidates from the 28 tested, and should be validated as DNA methylation markers for lung adenocarcinoma. These validations should consist of examining a sufficiently large number of new subjects representing both genders and all four major ethnic/racial subgroups in the United States (Whites of non-Hispanic and Hispanic descent, Blacks, and Asians), as well as early and late stage cancer. Such studies are currently ongoing. Our analyses of the present sample collection, which contains modest numbers of representatives from all these groups, is very encouraging as they suggest that the markers function independently of subject age, gender or ethnic subgroup, and are positive in early stage cancer.

The next step would entail the exploration of different methods to measure these markers non-invasively in early stage lung cancer patients. Potential "remote" media to be considered are sputum, bronchioalveolar lavage, and blood plasma, all of which we are in the process of collecting for examination. The ability of our four-marker panel to clinically detect lung cancer with high sensitivity and specificity will depend on many factors. A loss of sensitivity might be foreseen due to the small amounts of DNA shed into the blood of each patient, but at the same time, an increase in specificity might be expected if tumor DNA is shed more readily into the bloodstream than DNA from adjacent histologically normal tissue.

To our knowledge, CDKN2A EX2, CDX2, HOXA1 and OPCML constitute the strongest lung adenocarcinoma DNA methylation markers identified to date, and we are working on further evaluations of their potential with great anticipation.

## Methods

### Study subjects

Lung adenocarcinoma and when available adjacent non-tumor lung was obtained from archival paraffin blocks from 51 subjects who had been treated at three Los Angeles hospitals: the Los Angeles County Hospital, the USC University Hospital and the Norris Comprehensive Cancer Center. Clinical information was missing for 5 patients. Of the rest, 28 were male and 18 were female, 14 were White of non-Hispanic descent, 14 White of Hispanic descent, 11 were Black, and 7 were of various Asian origins. Ages ranged from 37–82 years old at time of surgery (median: 58 years old). For 32 of these cases, a separate paraffin block containing histologically verified cancer-free lung was available. These adjacent non-tumor lung samples were supplemented with 6 additional cancer-free archival samples from lung cancer patients for which the tumor block was unavailable, and 11 archival non-tumor lung samples from patients operated for non-cancer reasons, such as pneumothorax or emphysema. All studies were institutionally approved by the University of Southern California Institutional Review Board (IRB# HS-016041, HS-06-00447), and the identities of patients were not made available to laboratory investigators.

### Tissue samples and DNA extraction

Hematoxylin and eosin-stained slides were reviewed by an experienced lung pathologist (MNK) to support the original classification of the tumor and to select optimal tumor and non-tumor areas of the specimens. DNA was extracted from microdissected tumor and non-tumor lung samples via proteinase K digestion [35]. Briefly, cells were lysed in a solution containing 100 mmol/L Tris-HCl (pH 8.0), 10 mmol/L EDTA (pH 8.0), 1 mg/mL proteinase K, and 0.05 mg/mL tRNA and incubated at 50°C overnight. The DNA was bisulfite converted as previously described [13].

### DNA methylation analysis

DNA methylation analysis was done by MethyLight as previously described [14]. Primer and probe sequences were as described [14,36,37]. In addition to primers and probe sets designed specifically for the gene of interest, an internal reference primer and probe set designed to analyze Alu repeats (Alu) was included in the analysis to normalize for input DNA [38]. The percentage methylated reference (PMR) was calculated as the GENE:reference ratio of a sample divided by the GENE:reference ratio of *in vitro *methylated (*Sss*I-treated) human white blood cell DNA and multiplying by 100 [14]. Occasionally, PMR values over 100 were observed. This can happen when genes are very heavily methylated in the cancer sample, while the SssI-treated sample (in spite of extensive *in vitro *DNA methylation) is not fully methylated at that locus. This does not affect the significance of the loci identified in this study, as the same batch of *Sss*I-treated DNA was used throughout the study.

### Statistical analyses

PMR values of AD were compared to AdjNTL and NTL lung as continuous variables by means of the Wilcoxon rank sum test. For the comparison of paired AD and AdjNTL samples from the same patients, the Wilcoxon signed rank test was used. To control the false discovery rate at 5%, a multiple comparisons threshold was set. It was only applied to those 20 loci for which no previous information supporting a hypothesis of DNA methylation in lung AD was available [15]. Receiver operating characteristic (ROC) curves were plotted using the AD vs. all AdjNTL lung PMR values and JMP 6.0 software (SAS Institute, Cary, NC). The distribution of PMR values by group (AD, AdjNTL and NTL) were shown using log-transformed data in JMP 6.0. The two-dimensional hierarchical clustering was carried out using JMP 6.0 and log-transformed PMR values. VHL, which was negative in all specimens, was omitted from the clustering analysis. Associations between age, gender and race of AD cases were tested by dichotomizing the subjects either by the presence/absence of DNA methylation, or, if the samples were frequently methylated, by the median of all positive PMR values. All statistical tests were two-sided.

To determine which combinations of markers would be most effective to correctly identify tumor vs. non-tumor samples, we fit a random forest classifier to the data set, using the R programming language (v 2.5 [39]) and 87 samples and 28 variables (2 AD samples with missing PMR data were omitted, resulting in 49 AD/38 AdjNTL). Using bootstrap samples of the data, we grew a forest of 30,000 trees. Splits were determined using a random sample of five variables and trees were grown until there was only one observation in each leaf. We determined error rates using the observations that were not used to generate the trees. For each observation, its outcome was predicted by having the majority vote from the trees that were generated without the original data point in their bootstrap sample. These predicted values were compared against the true tissue type to estimate prediction error.

## Competing interests

IALO and PWL are shareholders of Epigenomics AG, which has a commercial interest in the development of DNA markers for disease detection and diagnosis. None of the work performed in the laboratories of the authors is or has been supported or directed by Epigenomics.

## Authors' contributions

JAT was involved in marker design, experimental execution and initial analysis. JSG was involved in experimental execution and extensive data analysis, drafting the manuscript, and generation of figures. KDS oversaw statistical analysis and drafted statistical sections of the manuscript. PWL provided experimental advice and mentoring for JAT. ST helped locate and section most of the tissues and provided the linked and de-identified clinicopathological information. WC provided additional samples through the Norris Cancer Center's Los Angeles area tissue discard repository. JAH provided non-cancer lung samples and statistical discussions. MNK reviewed all slides prior to microdissection. IALO designed the study, oversaw all aspects of the project, mentored JAT and JSG, and revised manuscript drafts. All authors reviewed and commented on the manuscript during its drafting and approved the final version.
